# Alveolar Abscess

**Published:** 1895-03

**Authors:** E. Herbert Adams

**Affiliations:** Toronto.


ARTICLE III.

### ALVEOLAR ABSCESS.

BY E. HERBERT ADAMS, M. D., D. D. S., TORONTO.

Read before the Toronto Medical Society.

Alveolar abscess is a term applied to any abscess having its origin in the alveolar process of either of the maxillæ. It is generally due to a pericementitis occurring at the apex of the root of the tooth, and caused by the death of the dental pulp. The first pus is pent up in the apical space by bony walls, and the pressure being very great results in the rapid destruction of the surrounding osseous tissue. The pus burrows where there is least resistance, and on account of the cancellous nature of the bone surrounding the root, and the denser nature of the bone nearer the surface, a larger pus cavity is formed.

The pericemental membrane surrounding the apex of the tooth is even yet not perhaps destroyed; but its fibres become elongated and their meshes filled with pus; the swollen tissue forming the shreddy bag-like mass so often seen attached to the end of a root of an abscessed tooth after extraction.

Should the outer lamina of bone be perforated, the pus has then ready exit through the soft tissues. The pain now lessens, and the symptoms abate somewhat. This may, however, be only a temporary cessation. The whole side of the face may swell up, the eye be distorted, or the jaws be so stiffened or swollen that they cannot be separated sufficiently for feeding purposes.

An examination will show a large swelling over the root. The swelling will usually show signs of fluctuation, and if left to itself will generally open just above the root. It is better, however, to anticipate nature by opening with a bistoury. After the discharge of pus, the swelling and pain usually subside.

Unless the affected tooth is removed, or the diseased pulp removed from the interior of the tooth by a dentist and the pulp cavity rendered aseptic, a chronic source of irritation is kept up, and the abscess assumes the chronic type.

In acute alveolar abscess there may be seen, occasionally, a considerable elevation of temperature, even as high as 103° or 104°. During abscess formation, and before the pus has found exit, a peculiar dull, throbbing pain is often present, and the lymphatics at the angle of the jaw are sometimes sore and swollen.

An abscess, if left to itself, usually assumes the chronic form, the pus continuing to be discharged, but in lessened volume. In the chronic form of alveolar abscess the burrowing of pus may cause a fistulous opening in the cheek, chin or neck, though the most usual place for abscesses to discharge is on the gum over the roof of the affected tooth.

Abscesses associated with the wisdom teeth, or third molars, sometimes pass in the direction of the parotid region. In these cases it is not uncommon to find the orifice of the fistula as low down as the clavicle. This is due to the unyielding character of the parotid fascia, which is a continuation of the deep cervical.

On account of the close relation of the roots of the teeth of the upper jaw to the antrum abscesses may open in the maxillary sinus, and thence be discharged through the nares. These cases are frequently mistaken for a diseased condition of the nasal passages, and treated accordingly, and, of course, invariably without success. The relation of the roots of teeth to the antrum is variable. In some cases the floor of the antrum is perforated by the first and second molars, so you can readily see how an abscess can open in this way.

The habit of applying hot fomentations, poultices and counter-irritants to the cheek and face is often to blame for abscesses pointing on the face and neck.

One of the most common places for abscessed teeth of the upper jaw to open on the face is just beneath the malar bone, and just in front of the anterior border of the masseter muscle. A disfiguring scar often results, if the abscess is allowed to open in this or any other facial region. Such abscesses may discharge anywhere below the region of the eye.

Occasionally, abscesses of the superior incisors discharge directly into the nasal cavity, and an abscess of an anterior tooth has been known to pass back beneath the mucous membrane of the hard palate, and discharge at the junction of the hard and soft palate.

The greater number of abscesses discharging on the face are in the lower jaw. This is probably due to gravitation. Frequently these abscesses open first on the gum, but during the healing process this opening becomes closed. Little pus perhaps remains as the abscess becomes chronic, but the slow burrowing of this, according to the law of gravitation, causes it finally to find exit through the lower jaw. There may be no pain nor other symptoms until this opening has occurred, much to the surprise and annoyance of the patient; the pus in some cases passing directly downward through the bone, but more frequently passing outward into the soft tissues, and then following these downward to point at the lower margin of the jaw.

Blind abscesses may occur, the pus being small in quantity, and being apparently absorbed without any external opening being formed for its exit.

Occasionally, alveolar abscesses have been known to cause extensive necrosis of the bones of the face. This is more especially the case in strumous or syphilitic patients.

Abscesses may also form osseous cysts on the side of the jaw. The pus, instead of being absorbed, is provided for by the expansion of the outer plate of the bone. These cysts form somewhat rapidly, and are sometimes half the size of a hazelnut. The rapid growth of these cysts is an important point in diagnosis.

In persons with an abnormally small jaw, the eruption of the wisdom teeth often cause severe inflammation and abscess, the jaw being too small to accommodate the new tooth. These abscesses generally discharge at the margin of the gum, but the swelling is often so severe as to cause almost complete immobility of the jaw. In a recent case where it was impossible to open the jaw sufficiently to extract the offending wisdom tooth, the second molar was removed, and of course the third molar had now a chance to come forward and the inflammation soon subsided.

Imprisoned teeth may be a cause of alveolar abscess. The diagnosis of these forms is rather obscure. A probe passed into the sinus, if one has been formed—if not, a bistoury passed through the softer parts and bone—will often assist in the diagnosis of such cases. Abscesses of temporary teeth require especial care, and if they are not amenable to treatment the diseased tooth should be extracted.

The *diagnosis* in most cases is comparatively simple; but from what has been said it will be seen that the diagnosis, of some forms at least, of alveolar abscess is not an easy matter for the general practitioner. And it is these unusual and anomalous cases that most frequently come under the observation of the physician and surgeon, and the ignorance displayed in treatment renders matters often uncomfortable for the patient, and not infrequently so for the surgeon.

A simple means of testing whether a tooth is abscessed is by rapping the tooth with an instrument. If it should prove tender on pressure, the apical pericemental membrane is inflamed, and the root of the tooth probably abscessed.

Abscesses most frequently occur on teeth with dead pulps. In such teeth the natural translucence of the tooth is gone, the dentinal matter, due to the disintegration of the pulp. The dark color and opacity is often very marked, but is occasionally so slight as to escape notice. If, however, the patient is placed in the sunlight and the rays of light reflected on the teeth by means of a mirror, a slight opacity will be noticed.

The patient's notice is often directed to a painful tooth as a possible cause of the trouble, but it must be remembered that neither a decayed tooth nor pain need necessarily be present. The pulp may have died from some other cause, and the diagnosis can be made by the opacity of a tooth and its tenderness on pressure.

The following are some of the cases which have been aecorded in dental and medical literature as cases of mistaken diagnosis:

Dr. Otto Arnold (*Dental Review*) mentions a case where a patient was confined to her bed by what her physician supposed to be diphtheria. After being treated for about a week, she began to suspect that her teeth were in some way implicated. The pharynx and tonsils were severely inflamed, but after some difficulty the diagnosis of an impacted wisdom tooth was made, and on its extraction she got well.

Dr. C. R. Butler (*Items of Interest*, 1891) mentions a case diagnosed by the patient's physicians as carbuncles, but which proved to be due to alveolar abscesses from three dead teeth. On their extraction a cure was affected.

J. P. Wilson (*Items of Interest*, 1888) mentions a case of alveolar abscess of eight years standing, where there was a fistulous opening over the clavicle, near the place of origin of the platysma myoides muscle. The disease had been pronounced by a council of physicians to be of a strumous character. He removed the roots of a diseased first molar and the discharge soon ceased, and the abscess healed without further treatment.

Dr. Tees (*Items of Interest*, 1886) mentions a case where death occurred from a surgeon performing a surgical operation on a case of alveolar abscess with much facial swelling, the patient sinking gradually after the operation. He states that the abscess differed in no way from an ordinary alveolar abscess, the three roots of the first left superior molar being diseased, and the swelling of a sudden and recent nature.

A case recently occurred which had the following history: A boy had a swelling of the face. His family physician applied poultices and hot fomentations to the side of the face, and afterwards opened into the pus cavity with a lancet. A running ulcer formed, which lasted for about a year and three months. The school authorities refused to admit the boy to school during this time on account of the disease. The boy was examined by a couple of physicians from the civic health department, who also recommended his detention from school. He would probably be, absent from school still and the abscess still discharging had not another physician made the diagnosis of an abscessed tooth. On the extraction of the tooth, the abscess healed readily, and the sinus closed of its own accord.

The *treatment* is comparatively simple in most cases, and consists in the evacuation of the contents of the pus cavity, and in injections of antiseptics until it is rendered thoroughly aseptic.

In the more simple cases this is readily accomplished by a dentist drilling through the root canal and thus allowing an exit for the pus, and an opening through which antiseptics can be injected. It is rare, indeed, that a skilled dentist cannot successfully treat even the worst cases by this means. Of course, if the offending tooth is for any reason considered of no value, the simplest method of cure is its removal, when the abscess will, as a rule, heal without any medication.

In some cases a simple way is to drill through the alveolus, just above the root of the tooth, and thus give an exit for the pus, and an opening for antiseptic medication. If it is desirable to keep the sinus open, a simple method is to place a pledget of cotton, soaked in a strong solution of carbolic acid, in the sinus.

In acute alveolar abscess where there is much swelling of the face, it is often well to endeavor to cause the abscess to point on the buccal surface of the gum over the root of the abscessed tooth. This can often be accomplished by the application of a counter-irritant, such as capsicum or cantharidine, to the gum overlying the root of the affected tooth. A roasted fig or raisin is said also to accomplish the same result.

All applications of hot fomentations, or poultices or counter-irritants to the external surface of the face should be religiously avoided, and if the abscess seems to have a tendency to point externally a free incision for the pus should be made *in* the mouth, and the counter-irritants or other medicaments applied *in* the mouth. This will prevent many an opening on the face, and its consequent scar.

Constitutional treatment should not be neglected where indicated. A saline cathartic will often assist in hastening the removal of an acute abscess.

In those cases where there is fistulous opening on the face, it is always well to direct, if possible, the discharge into the mouth. If this is done the sinus will heal of its own accord, and the abscess can then be treated in the usual manner. As a rule, the sinus needs little attention after the abscess has healed.

Often considerable disfigurement results from the scar due to a fistulous opening on the face. If under the chin, the scar does not show much, and in a male can be covered by a beard. If on the neck it may be hidden by the clothing; but on the cheek, especially in a female, little can be done to hide the disfigurement.

And here let me give a note of warning. A great many cases have come under my observation where surgeons have made external facial excisions simply because the abscess showed a tendency to point on the face. In one case where the simple extraction of the tooth would have been all that was required to heal the abscess (though even that was not necessary), a prominent surgeon made a crucial incision in the cheek, thrust in a couple of his fingers as an exploratory procedure, and then ordered a lotion of ac. carbol., 1 in 20, to be applied externally.

It is needless to state that such treatment is very reprehensible. There are cases where an external incision is indicated, but these are the exception and not the rule. In such cases, if the knife is used in a conservative manner, the healing process is speedy on account of the vascularity of the facial tissues, and often no preceptible scar remains.

From an æsthetic point of view, the surgeon should always endeavor to give vent to the pus by an incision inside the mouth, and not by an incision on the surface of the face.

One of the hardest tests to which my powers of argument were ever put was in persuading a fellow-practitioner from making a great gash in the cheek of a lady who had an alveolar abscess, which any good dentist could successfully treat by drilling through the root canal, and giving vent to the pus in that manner.

In regard to antiseptic medication any reliable antiseptic will do. Carbolic acid, peroxide of hydrogen, listerine and campho-phenique are perhaps the favorites with dentists. Thymol, creasote, oil of cloves, oil of cinnamon, sanitas, salicylic acid, iodine, and various other antiseptics, all have their advocates.

In this paper I have purposely omitted going into the detail of treatment of the simpler forms of alveolar abscesses, as they belong to the domain of the dentist and not of the surgeon. I would also strongly recommend that in all cases of doubtful diagnosis a competent dentist or oral specialist be called into consultation. These abscesses respond speedily to proper treatment, and the diagnosis, too, is, as a rule, simple to the dentist or oral specialist; and yet there are innumerable cases where the patient has been disfigured or inconvenienced for years by this disease simply through the ignorance of their family physician, who has failed to make a correct diagnosis. Later on, perhaps, a correct diagnosis is made by a dentist, or someone who is familiar with the disease, and the patient is cured in a few days or a week. Naturally, the patient is much embittered against the medical man whose ignorance allowed such a foul ulcer to remain on their face for such a long time.

Prophylaxis is of importance in the prevention of alveolar abscesses, but this belongs largely to the domain of the dentist. It should, however, be the duty of every physician, whenever he finds decayed or offensive teeth present in any patient, to impress on them the importance of visiting their dentist and having their teeth attended to. —*Dominion Dental Journal.*

